# A Traditional Chinese Medicine Traceability System Based on Lightweight Blockchain

**DOI:** 10.2196/25946

**Published:** 2021-06-21

**Authors:** Zhengfei Wang, Lai Wang, Fu'an Xiao, Qingsong Chen, Liming Lu, Jiaming Hong

**Affiliations:** 1 School of Medical Information Engineering Guangzhou University of Chinese Medicine Guangzhou China; 2 Shenzhen Ant Network Service Co LTD Guangzhou China; 3 Clinical Research and Data Center, South China Research Center for Acupuncture and Moxibustion Medical College of Acu-Moxi and Rehabilitation Guangzhou University of Chinese Medicine Guangzhou China

**Keywords:** blockchain, traditional Chinese medicine, TCM, traceability system, fake drugs, IPFS, fraud, traceability

## Abstract

**Background:**

Recently, the problem of traditional Chinese medicine (TCM) safety has attracted attention worldwide. To prevent the spread of counterfeit drugs, it is necessary to establish a drug traceability system. A traditional drug traceability system can record the whole circulation process of drugs, from planting, production, processing, and warehousing to use by hospitals and patients. Once counterfeit drugs are found, they can be traced back to the source. However, traditional drug traceability systems have some drawbacks, such as failure to prevent tampering and facilitation of sensitive disclosure. Blockchain (including Bitcoin and Ethernet Square) is an effective technology to address the problems of traditional drug traceability systems. However, some risks impact the reliability of blockchain, such as information explosion, sensitive information leakage, and poor scalability.

**Objective:**

To avoid the risks associated with the application of blockchain, we propose a lightweight block chain framework.

**Methods:**

In this framework, both horizontal and vertical segmentations are performed when designing the blocks, and effective strategies are provided for both segmentations. For horizontal segmentation operations, the header and body of the blockchain are separated and stored in the blockchain, and the body is stored in the InterPlanetary File System. For vertical segmentation operations, the blockchain is cut off according to time or size. For the addition of new blocks, miners only need to copy the latest part of the blockchain and append the tail and vertical segmentation of the block through the consensus mechanism.

**Results:**

Our framework could greatly reduce the size of the blockchain and improve the verification efficiency.

**Conclusions:**

Experimental results have shown that the efficiency improves compared with ethernet when a new block is added to the blockchain and a search is conducted.

## Introduction

In current society, drugs are an important guarantee of healthy human life. Contrastingly, counterfeit drugs cannot cure diseases and may cause physical damage to patients. In the worst cases, serious drug accidents even lead to death. According to World Trade Organization statistics, thousands of patients have died of drug accidents due to ingestion of counterfeit drugs worldwide [1]. Drug accidents are more likely to happen in developing countries, resulting in substantial medical economic losses.

For the general population and even for physicians, it is difficult to identify counterfeit drugs; owing to this difficulty, it is more difficult to prevent the use of counterfeit drugs. The circulation of traditional Chinese medicine (TCM) must go through a series of processes, from cultivation, fresh product collection, processing, and circulation of prepared pieces to their sale as TCM patent medicines [2]. Because the composition of proprietary Chinese medicines is complicated, drugs are not easily identified by the most direct senses. To effectively prevent counterfeiting of drugs and to crack down on harmful behaviors in all aspects of production, it is important to establish a reliable drug traceability system. From the planting of drugs at the base and their growing, harvesting, processing, packaging, storage, and circulation, to selling to patients, freshly collected products must go through many different processes before becoming patent medicines, such as prepared pieces. Every link of the drug circulation process should be tracked and recorded; blockchain can implement each process of every producer and seller to achieve traceable destinations and traceable sources.

Blockchain can help patients understand the past life path of a drug. Therefore, if there is a problem with the quality of the drug, it can be traced back to the source of the problem.

The United States and China have adopted the “whole process traceability” mode [3,4]. Drug traceability includes the production, circulation, use, and other aspects of drugs. This mode realizes gradual transmission and traceability of drug information and achieves interlocking links. It is conducive to preventing counterfeit and inferior drugs from entering the drug supply chain, standardizing the drug circulation order, and ensuring drug safety. The European Union has established a unified European drug certification system, which is composed of the National Drug Certification Systems of Member States, European Drug Coding Center and European central hub [5].

In May 2011, the Ministry of Commerce of China, the State Administration of Traditional Chinese Medicine, and the State Food and Drug Administration (SFDA) carried out a pilot project of a Chinese medicine traceability system in Chongqing. In 2015, the State Administration of Traditional Chinese Medicine took the lead in supporting “quality traceability,” and they proposed quality control standards for the whole process of TCM production and the standards and evaluation system of key TCM products with quality information traceability [6]. In 2018, the SFDA promoted the construction of a drug information traceability system, realized “one item, one code, and the same tracking,” strengthened the exchange and sharing of traceability information, and realized whole product and whole process traceability [7]. In 2019, the SFDA issued the *Guidelines for the Construction of Drug Information Traceability System*, *Drug Traceability Code Coding Requirements*, the *Basic Technical Requirements for Drug Traceability System*, and other informatization standards [8].

However, traceability systems pose great security risks. Because the drug production data of each link are stored in the system, these data could be easily tampered with, deleted, or denied. If such tampering were to occur, the authenticity of the traceability system would not be guaranteed, and the system would lose application value. The emergence of blockchain technology fundamentally changed this situation. Blockchain is a type of decentralized distributed account book with characteristics that cannot be tampered with, deleted, or denied. Storing traceability information in the blockchain prevents the abovementioned risks and ensures data security.

At present, blockchain has produced a new batch of technical solutions which have been implemented in clinical trials in some medical institutions [9]. Blockchain provides great convenience for the distribution management of medical data and the sharing under the control of access rights. Blockchain’s tamperproof and facile query characteristics can promote its use with medical resources.

However, these blockchain systems based on drug systems have the following problems:

1. Data explosion. As a chain record, the blocks store all the data in the drug transaction from beginning to end, and the data storage creates a heavy load on the system.
2. The low efficiency problem. The characteristics of the chained records will affect the query speed; moreover, the data capacity of blockchain is very large, which will also affect the query efficiency.
3. Security issues. Blockchain originates from Bitcoin; however, Bitcoin is not suitable for the medical field. Medical data are only disclosed to the public on the premise of ensuring security and privacy. Blockchain has distributed and multicenter characteristics. However, it is also necessary to ensure that important system data are not stolen and to prevent malicious users from attacking the data. Therefore, previous systems require improvement.

In view of the drug traceability development needs and the deficiency of blockchain applications, this paper proposes a new TCM drug traceability system based on blockchain that effectively protects data security, fundamentally realizes information traceability, and prevents data tampering and denial.

The main contributions of this work are as follows.

First, we propose a Chinese medicine traceability system architecture based on blockchain and the InterPlanetary File System (IPFS) to effectively solve the blockchain information explosion problem. In the design of the blocks, the blocks are divided horizontally, and the header and body of traditional blockchain are separated. The header is stored in the block, and the body is stored in the InterPlanetary File System (IPFS). The design achieves the effective verification of information but also reduces the block size. Further, we divided the blocks vertically. The length of the blockchain increases with the time dimension; this increase will affect the verification efficiency of the blockchain. Therefore, we selected a threshold (time or size) to segment the blocks. When users verify new blocks, they only need to copy certain parts of the blockchain.

Second, we evaluate the performance of our proposed architecture based on blockchain and the IPFS. Compared with the Ethereum network, our experiments demonstrate that our proposed architecture outperforms the Ethereum network in terms of the time cost for processing ledger update and query.

Third, in the initial stage, a blockchain is added to the original drug traceability system to ensure that the information will not be tampered with or denied. This is not a substitute but a supplement for the system. After it gradually becomes mature, the new TCM herbal medicine traceability system will be transplanted to the blockchain to maintain the smooth transition.

## Related Work

Due to its decentralized and tamperproof nature, blockchain has received extensive attention in many areas, especially in food and drug traceability, as well as for electronic health record (EHR) sharing and traceability. However, there are few research results on the traceability of TCM.

### Food and Drug Traceability Based on Blockchain

In 2016, Tian et al [10] developed a new blockchain system used radio-frequency identification (RFID) to improve the efficiency of collecting information automatically with a corresponding new system for storage and management. Galvez et al [11] proposed the use of blockchain to prevent economic losses and erosion of consumers’ trust after viewing its potential in traceability and authenticity. Galvez et al indicated that different cases are operable, including plant food, animal food, and other industries, on the basis of existing products.

Toyoda et al [12] used smart contracts in data management and made changes to enable the smart contracts to be classified by levels; this smart contract level classification improved the management efficiency. In real-world data exploration of supply chains in IBM and Tsinghua University’s collaboration work [13], IBM tested the time cost of uploading data in a public chain and showed that data exploration decreased the efficiency.

Daniel Tse et al [14] prospected a specific food supply chain blockchain in 2017; soon, San Miguel et al [15] raised concerns regarding the food supply chain, integrating agricultural information technology in traceability.

Blockchain has also been developed in drug-related areas. In Europe, a biobank was built for more comfortable data management, including medical data related to drugs. N Mamo et al [16] proposed a biobanking scheme called Dwarna for dynamic consent.

### EHRs Based on Blockchain

Traditional electronic medical records (EMRs) have been gradually transformed into EHRs; this new type of digital record enables patients and physicians to manage the patient’s health record together rather than allowing only the physician to control all the data, as before. This progress increased the convenience and efficiency of medical service; however, a large problem remains, namely that patients and physicians’ privacy can also more readily be violated [17]. With new cloud computing technology, Dubovitskaya et al [17] used smart contract blockchain technology with new cloud computing technology for access control to guarantee high security levels.

With mobile phones, access to medical data is becoming increasingly convenient for users, including both patients and physicians; however, the tasks of uploading and sharing the data are challenging. Mobile devices can detect people’s health information and share it in the cloud; however, this requires a cloud server solution that is honest-but-curious [18]. Hang et al [19] proposed a new blockchain platform for patients to access their records in a comprehensive and immutable way to meet security policies; a case study of Hyperledger Fabric and a benchmark study were used in real hospitals, and these studies revealed this platform’s potential for acceleration.

In addition to the medicine protection area, C Krittanawong et al [20] introduced a blockchain application in the area of artificial intelligence; technology by Tezos used blockchain in robotic event registration and verification, and Atheon's platform used blockchain for time-resuming reduction. Retrievable information, automated uploading in lack for robots marketplace, and global transparency are provided by blockchain [20]. In some traditional work, the abovementioned problems could not be readily solved; for example, in Wu et al’s [21] work, they provided total access to a cloud server but could not promise safety or authenticity of the EHRs.

Because in the work mentioned, total access to cloud severs was given, the researchers changed the initial structure of Bitcoin to avoid this weakness [22]. They used smart contracts for access control to defend against attackers. Also, smart contract and blockchain were used in data management. The architecture consisted of data offloading and data sharing through access control by verifying a public key to find the corresponding ID with the IPFS data storage system. Unlike pioneering studies, this paper describes an experiment on a proposed system with two virtual machines as miners, two as the administrator and EHR manager, IPFS on Amazon, and Rivest-Shamir-Adleman (RSA) encryption. Appropriately designed experiments afforded better performance.

### Lightweight Blockchain Research

Ismail et al [23] described how a combination of distributed ledger (DL), consensus protocol, and cryptography technology can be used for blockchain updating. They proposed a blockchain-based health care data management architecture after considering different parts in which the head blockchain manager regulates the network and block transaction work mode. Changing blockchain nodes to decrease the burden, they used a method called a canal to collaborate with network participants. The architecture included a ledger; notification manager records; agreement consensus, such as the practical Byzantine fault tolerance consensus protocol, in proof of work (PoW); and data replication controlled by a strong consistency model and constant prefix consistency.

A new blockchain architecture must guarantee the safety of the blocks in the arrangement of nodes and ledgers during updating and query. In denial-of-service (DoS) and time-based attacks, it should be impossible to modify or attack the data. To address efficiency and energy cost problems, clusters for different hospital nodes with a manager maintaining the ledgers could be helpful [24]. Arjona el al [25] adopted a lightweight fingerprint recognition solution; their QFingerMap16 method can be implemented in low-cost sensor nodes using lightweight dual-factor protocols. In work by Arjona et al [25], a sensor communication protocol was implemented to defend against remote attacks and impersonation attacks.

One of the main concerns in research on the use of blockchain with health care data is effectively decreasing the update time. Lwin et al [26] built a lightweight trust system with a consensus algorithm; their scheme distributes nodes in a mobile ad hoc network to create a tamperproof and optimized link state routing protocol. Fu et al [27] used an interleaving encoding algorithm to propose a lightweight message sharing scheme. This scheme applies an interleaving encoder to conceal sensitive information. EMRs are mapped to *n* different short shares, and the shares are transmitted to different nodes on the blockchain. Fu’s sharing scheme reduces the cost of shorter shares and provides an efficient reconstruction process; moreover, the use of indexes increases the system stability [27].

### Current Medical Blockchain Products

Most well-known medical blockchain products use advantages of blockchain such as decentralization, immutability, security, and transparency, including solutions for third-party trust, prevention of data tampering or deletion, cryptography, data management, and data tracking and storage problems.

The most well-known blockchain products are MedRec [28], MedicoHealth [29], MeFy [30], and MediBloc [31]. Based on Bitcoin, the medical decentralized distributed ledger technology MedRec was proposed as a solution for system interoperability and provided access for more clients to manage their medical records, such as patients and physicians, with smart contract and consensus. Three types of contracts are included in MedRec for real-time sharing. A summary contract provides hashes for safety [32].

Similar changes were designed in MedicoHealth, MeFy, and MediBloc. MeFy promised to provide physicians with entry access and to protect patients' privacy and anonymity. MediBloc aids the reuse of old medical data history to provide more choice on similar occasions when medical references are needed [33]; they proposed a scheme for drug history queries for physicians, nurses, patients, pharmacists, and management to promise safety and efficiency of the supply chain.

Kuo et al [34] suggested a basic structure in which a Merkle tree prunes the transactions in the tree. The root and header of the tree were designed for easy verification and safety so that an attacker could not undertake the cost of recreating blocks. Ethereum, Hyperledger, and MultiChain are additional emerging applications of blockchain that adhere to standards of the health informatics community. The PoW consensus protocol has been widely adopted. Zcash, Dash, and Monero focus on privacy or anonymity of transaction information for users such as patients.

Many distributed ledger technology (DLT) platforms were examined in [35], such as Bitcoin for digital currency conducted by consensus algorithm; Ethereum virtual machine using smart contracts; Multichain, which uses an application programming interface (API) and command line interface that are easy to interact with to maintain and deploy DLT systems; and EOS, a widely known delegated proof-of-stake cryptocurrency platform with an algorithm consisting of 21 block producers. Cardano supports smart contracts and decentralized applications using a Follow the Satoshi algorithm to introduce a certain amount of randomness without relying on any PoW consensus algorithms. Also, there are many Hyperledger medical blockchain platforms, such as Hyperledger Fabric, which is constructed within the Hyperledger ecosystem with a modular design employing an identity layer and channel layer architecture supported by simplified Byzantine fault tolerance; Hyperledger Sawtooth, which uses a proof-of-elapsed-time algorithm that depends on Intel Software Guard Extensions under a new type of trusted execution environment; and Hyperledger Burrow. Additional platforms use consensus-building and new permissions for verification and security with different data structures, such as Directed Acyclic Graph; these platforms include IOTA, Corda, and Waltonchain. Our research specifically focuses on Ethereum.

## System Requirements and System Architecture

The length of the blockchain itself is difficult to decrease; an excessively long chain can influence the transparency efficiency. Lightweight blockchain divides some data from traditional blocks. Lightweight blockchain uses file systems to decrease the initial data storage scale. Notably, because the blocks are decreased, the chain is also cut shorter. The new chain in the lightweight structure is lighter than that in traditional blockchain. After the new file system is introduced, the search efficiency controlled by the traditional data scale can be improved because the chain is shorter. In this paper, we used a lightweight design.

### System Requirements

#### Overall Requirements

In a TCM traceability system, generally speaking, the system should meet the following requirements: realizing the whole traceability process of drugs from planting and processing to distribution to hospital to patients; provide lightweight blockchain to prevent data explosion; ensure the safety of the blockchain to protect the privacy information of all categories of users; and increase the response speed of the blockchain, enabling users to trace authorized information.

In a private chain that provides authorized users with access for verifying blocks, the information security and reduce network pressure must be protected.

#### User Requirements

In a TCM traceability system based on blockchain, there are four types of users: enterprises (eg, planting enterprise users, production enterprise processing users, sales enterprise users), hospitals, patients, and regulatory departments.

For the enterprise, the system should meet the following requirements:

1. The enterprise should have access to view the basic information of the drugs (excluding privacy) at all stages.
2. The sensitive information of the enterprise should be protected and not be leaked in the system.

For the hospital, the system should meet the following requirements:

1. Users are able to view the drug planting and processing situation.
2. If problems are identified with the drug, the information provided in the traceability system is provided as legal evidence.

For the regulatory authorities, the system should meet the following requirements:

1. When the authorities view all the data in the system, the data are equivalent to the traditional DBA.
2. The authorities can verify that the data in the system are legal and effective.
3. If there is a problem with a drug, the system should provide accountability according to the system information.

For patients, the system should meet the requirement that the patients can view the planting, processing, and sales information of the drugs they purchased.

If there is a problem with the drugs, the information provided in the traceability system can be used as legal evidence.

The end users are mainly government staff members controlling the safety and normal transaction of drug trades. The representative government end users are law enforcement personnel, such as SFDA and health commission employees. In China, these data should be administrated by the National Health Commission of the People’s Republic of China.

### System Architecture

**Figure 1:**
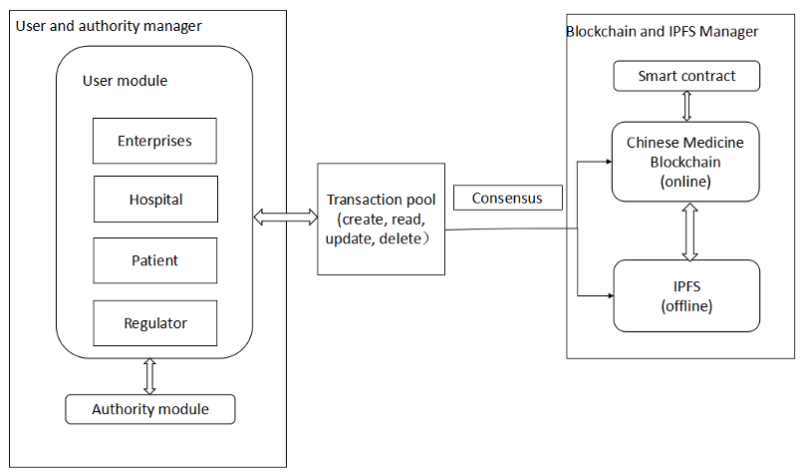
The architecture of the Chinese medicine traceability system based on blockchain.

As shown in Figure 1, the TCM traceability system based on blockchain consists of three components and their relationships:

1. User module and authority module. The private chain is used to register, manage and authenticate the users who join the blockchain. The authenticated users have different permissions on the system.
2. Transaction tools. The user activities, such as adding, deleting, modifying, and checking, generated in the TCM process are stored in the transaction pool, and these activities will be added to the blockchain and the IPFS.
3. Blockchain and IPFS manager. A certain number of activities in the transaction pool are selected in chronological order. After hashing and packaging, these activities are added to the blockchain and IPFS under the consensus mechanism action. Blockchain mainly stores the Merkle tree and signature of these activities; detailed TCM information is stored in the IPFS. This method avoids information explosion and prevents disclosure of private drug information.

#### User and Authority Manager

The module consists of two submodules: user and authentication. Users consist of enterprises (planting enterprise, production enterprise, processing enterprise, and circulation enterprise), hospitals, patients, and regulatory departments. Due to the private chain mode, users must be authenticated to participate in the blockchain's activities. The authentication module is equivalent to the certificate authority, which provides effective authentication for users joining the blockchain network. Our system not only stores all types of transactions generated by users, such as information related to the planting enterprise for planting of certain medicinal materials, but also stores the information related to modification and deletion of medicinal materials by users in the transaction pool.

#### Blockchain and IPFS Management

In Bitcoin, all nodes need to copy the entire blockchain to the local system (all the nodes maintain a replicated copy of the ledger). As the number of blockchain accounts increases, the following problems will arise:

1. Space: the storage space that nodes need to provide increases with the increasing number of account books.
2. Network: each node needs to download the account book, the network cost increases sharply.
3. Calculation: the calculation cost of the nodes in the verification process increases.

**Figure 2:**
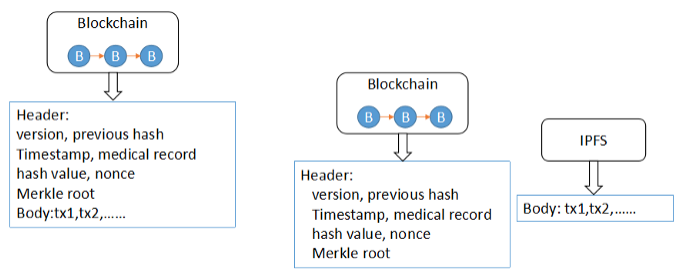
Blockchain structure. (A) Traditional blockchain structure. (B) Horizontal blockchain structure. IPFS: InterPlanetary File System; tx: text.

To solve this problem, we have improved the blockchain in two aspects. First, horizontal segmentation of blockchain was adopted. The traditional block, as shown in Figure 2, consists of two parts: a header and a body. The header stores the Merkle tree, timestamp, previous hash, and other parts. The header is mainly used to verify the correctness of the transactions. The body stores the packaged specific transactions; this part occupies most of the space of the blockchain. In the improved blockchain, only the header in the original block is saved, while the body is stored in the IPFS; this design greatly reduces the size of the blockchain.

**Figure 3:**
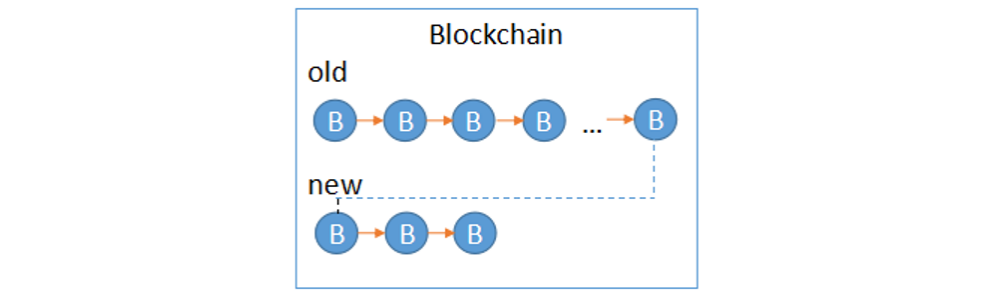
Vertical blockchain structure.

Second, vertical segmentation of the blockchain is adopted. The blockchain is formed with blocks according to the time sequence. As the time increases, the length of the blockchain increases correspondingly; this will lead to a sharp increase in the storage and calculation costs of the nodes. The improved blockchain is divided into two segments, as shown in Figure 3.

The new blockchain only stores the blocks added recently, and the old segment stores the past blocks. New and old blocks are connected by pointers. When nodes verify the newly added blocks, there is no need to copy all the blockchains; instead, only the new segment blocks are copied.

This approach greatly improves the verification efficiency and system performance. New and old blocks can be designed to be segmented according to time or size. If time is rotated as the threshold, the blocks are segmented by month, quarter, or even year according to the actual situation. Once the time node is reached, the old blocks generated are stored in the IPFS, and the new blocks are added to the new blockchain.

### Transaction Pool

This module has two functions: first, the information is generated by various users (eg, enterprises, hospitals, and patients) in the user module. Second, the miner selects a certain amount of transactions from the transaction pool and adds them to the blockchain.

Once these transactions are verified by the consensus mechanism, they are added to the blockchain and the selected transactions are deleted.

## System Implementation

The TCM data were collected from participants in the drug trade, including farmers, companies, and drugstores; two popular representative drugs were chosen for testing. The data contained each step in the drug production process. The data were generated from these parts: drug planting at the base, growing, harvesting, processing, packaging, storage, circulation, and selling to patients. The data were generated from these parties: TCM dealers, patent TCM pharmaceutical production companies, and raw material sources. The TCM dealer data consisted of the price, approval number, shop name, province, patent TCM pharmaceutical production medicine name, TCM medicine under quality control, and date of sale. The medicine enterprise data consisted of the names of the medicinal products, main raw materials, production enterprises, regions, and TCM medicine under quality control. The raw material source data included the names of the production companies, province, scope of business, and herbal TCM medicine under quality control. A total of thousands of pieces of data based on two types of herbal TCM medicine in circulation were used in our experiments.

**Figure 4:**
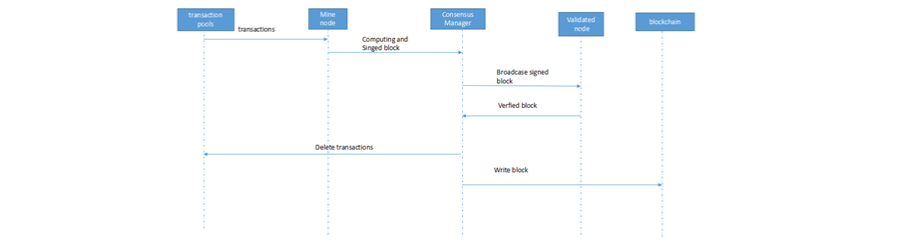
Data flow of the blockchain and IPFS manager. IPFS: InterPlanetary File System.

Figure 4 shows how the activities in the transaction pool are added to the blockchain and IPFS. Blockchain nodes are divided into mining nodes and verification nodes. All users in the blockchain are used as mining nodes or verification nodes:

1. Mining node (equivalent to miners): after mining starts, the transactions in the transaction pools (also stored in the database) are organized to form a pending block; this process is a hash calculation that conforms to the nonce rule. Once the calculation is successful, the block is submitted to consensus management and the blocks submitted by other miners are rejected by the consensus. Then, consensus management starts consensus processing and publishes an announcement to enable verification of the verification node. If the block is successfully verified, it is added to the blockchain.
2. Verifying the nodes. After receiving the announcement issued by consensus management, the pending transaction block is verified (this verification is very fast) and signed, and it is then sent to consensus management. According to consensus management statistics, if the threshold value (2/3) for joining the blockchain is reached, the header and body of the block are added to the blockchain and IPFS respectively, and the transactions that have been added to the block are deleted from the transaction pool. If this process is successful, the block joins the blockchain.

Our system, the smart contract empowered system, is implemented on the open source Ethereum platform. This platform includes five parts: the blockchain, IPFS (version 0.4.13), smart contract, Ethereum decentralized application (DAPP) platform and the third-party MetaMask wallet. The Ethereum DAPP application platform is composed of Node.js (version 8.9.4) and Truffle (version 5.1.12). It is implemented with the Truffle project framework, mainly through calling the relevant API to connect each part and import the relevant blockchain account information, compile and deploy the smart contract, and complete the relevant user requests. The smart contract code is written in solid language, and the Truffle contract API is used to synchronize transactions and deploy to the chain. The main function of a smart contract is to code according to the business rules of different identity registrations, traceability information upload, authorization and revocation of related roles, and query and access of end users, as well as to manage the corresponding permissions. The IPFS is mainly responsible for storing the encrypted privacy information of the Chinese herbal medicine supply chain. The IPFS performs synchronization of the data of the nodes and returns the final hash routing address. Finally, the blockchain is responsible for storing the smart contract code, completing the call of the relevant smart contract interface, packaging the execution result of the request in the form of the blocks, and writing the transaction records into the ledger after the node consensus verification.

A smart contract in Ethereum is deployed by means of a transaction by spending a certain amount of ether, the “cryptocurrency” of the Ethereum network, and invoked with input data via a transaction. In our system, the transaction is related to a TCM drug. The smart contract code is written in solidity language (version 0.4.21).

Details of the algorithms of traceability information data upload, authority control of traceability, and inquiry and access of traceability are provided in Textboxes 1-3, respectively.

**Algorithm 1: traceability information data upload.**

**Input:** TCM herbal medicine information, Blockchain length threshold L

**Output:** The hash returned by the InterPlanetary File System (IPFS), the block upload success information

**Acquisition phase**

1. A unique identification is assigned for a single traditional Chinese medicine (TCM) herbal medicine or batch by radio frequency identification tag, and the labels are sent to manufacturer A’s database.

**User registration**

2. The node initiates the registration information request.

3. The blockchain account address information and public private key pair are generated.

4. The smart contract-registration interface is called.

5. The enterprise deposits the initial transaction amount to the blockchain account address.

**Upload request**

6. The nodes input the TCM transaction.

7. The private key signs the request, and the encrypted information is stored in the IPFS.

8. The corresponding hash is returned.

**Blockchain nodes verification**

9. The traceability information attribute and encrypted information hash value are passed in as parameters.

10. The smart contract is executed and broadcasts the unique identification code of the bound enterprise, encrypted information hash value, and traceability information attribute.

**Calculation of the length of the blockchain**

11. The blockchain is divided into old and new parts, and the contract execution results are written to the new blocks.

**Return**

12. The blockchain upload success information is returned.

**Algorithm 2: authority control of traceability.**

**Input:** Traceability user and authorization information attributes

**Output:** Success tips

**Authorization:**

1. The user inputs the corresponding traceability user and authorization information attributes.

2. The user initiates the authorization request and encrypts the request with the private key.

3. The Ethereum application platform requests the blockchain nodes to call the smart contract authorization interface.

4. The request is broadcast to other nodes synchronously and the authorization information is written into the account book records.

5. The Ethereum application platform uses a public key to encrypt the corresponding key.

6. The user decrypts the key through the private key.

**Algorithm 3: inquiry and access of traceability.**

**Input:** The unique identification ID, traditional Chinese medicine (TCM) medicinal material identification ID and traceability information attribute of the traceability information owner enterprise

**Output:** The hash value of successful prompt corresponding to the query information, encrypted privacy traceability information

**Results**

1. The user inputs the unique identification ID, TCM herbal medicine identification ID, and traceability information attribute of the owner of the traceability information.

2. Ethereum extracts information attributes from the request interface and executes the contract.

3. Ethereum sends a request query to the InterPlanetary File System node and returns the encrypted privacy traceability information.

**Return**

4. The results are returned.

## Performance Evaluation

The construction of the TCM traceability system is mainly based on the Ethereum platform to build a public blockchain and private chain blockchain. Four Ubuntu cloud servers with the Geth client were built as different account addresses using Go language; the creation block information and Geth configuration files of the four nodes were configured, and Ganache-cli was used to initialize accounts and generate public and private key pairs. Four server nodes were formed into blockchain network nodes, and the underlying consensus trading environment was achieved. The Ethereum platform was built with the Truffle framework, and the Remix integrated development environment (IDE) visualization tool was used to perform the corresponding account address entry, compilation and deployment of the smart contract, and method call of the smart contract interface. IPFS file storage was mainly achieved by writing Web3.js script in the Truffle framework and calling the IPFS API. The core file of the whole framework was mainly Web3.js script. The core contained a smart contract address, IPFS calling interface, related function methods, and page code. The Geth + Truffle + IPFS terminal combination constituted the software environment of the traceability system. The hardware environment mainly consisted of a notebook computer, Core i7 processor (Intel Corporation), 16 GB memory, and 1 TB hard disk space. Three parts of the time and space capabilities of the system were tested: (1) the relationship between the storage space of the blockchain traceability system, IPFS, and new TCM traceability system with the upload times; (2) the relationship of the changes in the upload response time with the block capacity; and (3) the relationship between the changes in the query response time and the block capacity.

The results of these three time and space capacity tests showed that the processing level of the new TCM traceability system increases in time and space. The results were analyzed to further verify the comprehensive ability of the system.

**Figure 5:**
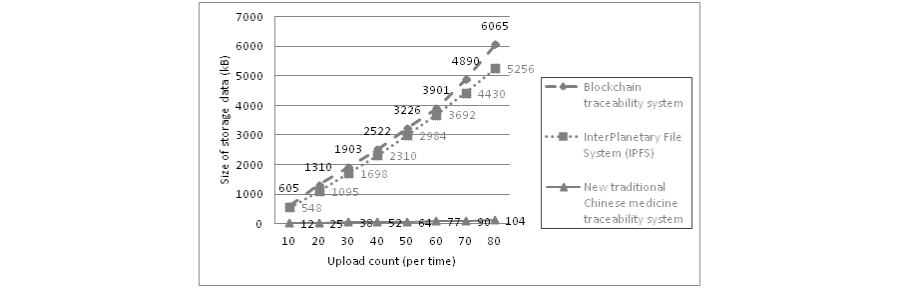
Comparison of the increase of the storage space according to the size of the data upload.

We uploaded the same traceable drug file multiple times in three different systems. Figure 5 reveals that although the common blockchain system has consensus traceability ability, the data on the chain will continue to increase steadily; this will eventually lead to performance problems and data security problems due to a long chain and data volume oversaturation. The storage space of the IPFS system increases steadily. The generalized Merkle directed acyclic graph structure endows the system with unlimited storage space. The IPFS structure will partition the block according to the file size and finally return the hash address. The new TCM traceability system combines the advantages of the common system and IPFS. The new TCM system greatly reduces the data storage capacity on the chain. When the space resources are optimally used, security problems caused by the data explosion on the chain are also prevented. Storing the business data on our system’s chain in a third-party distributed database further improves its information traceability and privacy protection abilities.

**Figure 6:**
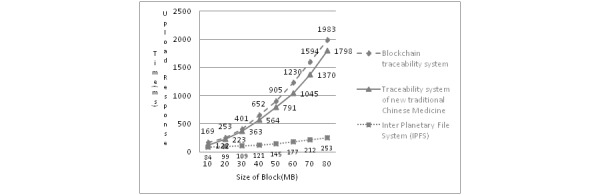
Comparison of the upload response times with different sizes of block storage.

Figure 6 reveals that the IPFS system has a strong upload response capability. The ability of IPFS to deal with bandwidth problems caused by the block capacity increase is also relatively obvious. The common blockchain traceability system obviously affects the response speed of the upload requests due to the continuous growth of the block capacity, and the response time will increase exponentially with the rapid growth of the block capacity. This increase creates response delay and affects the transaction writing process. Therefore, the new TCM traceability system improves the upload response speed of the system by combining it with IPFS technology and makes full use of the data distribution ability of hash tables to improve the user traceability effects. Due to the impact of the bandwidth performance and the complexity of the business logic code calling interface, the upload time is slow, so the efficiency does not reach the maximum; however, the overall optimization trend is obvious.

**Figure 7:**
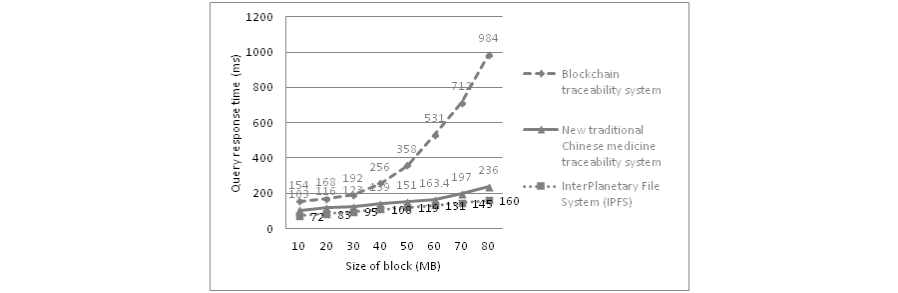
Comparison of the query response time changes with different sizes of block storage.

Figure 7 reveals that the query response time of the general blockchain traceability system is greatly affected by the block capacity. As the newly added transaction data are continuously written into the blockchain ledger, the number of blocks on the chain increases. When the query traceability information interface is called, the blockchain will search for data on the chain step by step; however, if the data have been hashed to form a Merkle tree structure, the response time becomes slower. The query ability of the IPFS system is very strong and stable. The new TCM traceability system combined with IPFS technology can greatly improve the response performance. Because the system only needs to store the hash address returned by the IPFS network, it controls the amount of business data on the chain to the minimum level, thus reducing the recursive query time of the system and obtaining the Merkle tree hash value; therefore, users enjoy the simplest and fastest information traceability experience.

## Discussion

Blockchain is a type of tamperproof distributed accounting technology. Blockchain possesses natural advantages when applied to the traceability of TCM herbal medicine. However, compared with the existing Bitcoin and Ethereum blocks, there is a much larger amount of information related to TCM herbal medicine, leading to a much higher risk of data explosion. In addition, some privacy information regarding TCM herbal medicine must be protected. In this paper, we designed a decentralized system based on blockchain and IPFS. Horizontal and vertical segmentation are conducted in the blocks to greatly decrease the data in our system; these segmentations solve the data explosion problem and protect privacy information.

The users of related drug administration input the unique identification ID, TCM herbal medicine identification ID, and traceability information attribute of the owner of the traceability information for the query system. Then, Ethereum extracts the information attributes from the request interface and executes the contract. After Ethereum sends the request query to the IPFS node and returns encrypted privacy traceability information, the results are returned. Administrative organizations such as the SFDA can obtain all the information they need to achieve chain data traceability for further investigation.

An experiment was conducted to verify the efficiency of the new method, and our method was also compared with other methods (Table 1).

**Table 1:** Performance comparison chart of the new TCM traceability system and current mainstream traceability systems.

Characteristic	Centralized traceability system	EPCIS^a^ network service traceability system	Blockchain traceability system	New TCM^b^ traceability system
Information traceability ability	Low	High	High	High
Tamperproof capability	Low	Low	High	High
Privacy protection capability	Low	Low	Low	High
Data dispersion capability	Low	Low	Low	High
Chain data size	N/A^c^	N/A	Large	Small
Data response speed	N/A	N/A	Medium	Fast

^a^EPCIS: Electronic Product Code Information Services.

^b^TCM: traditional Chinese medicine.

^c^N/A: not applicable (incomparable).

The data were collected from aspects of the drug trade for computer validation. Compared with Ethereum, our experimental results demonstrated that our proposed architecture shows superior performance in the following aspects:

1. The speed of uploading data to the blockchain is faster.
2. The speed of querying the blockchain improves.
3. Network traffic is improved because users only need to copy part of the blockchain.

In the future, we would extend the proposed work in the following aspects:

1. User node optimization: in this paper, transaction information is stored in IPFS, which may affect the system performance to a certain extent. In future work, we would add some super user nodes to store not only old blocks but also some transaction information.
2. Blockchain query optimization: as the blockchain length increases, the query efficiency decreases sharply. Therefore, a structure similar to an index would be designed in future work to accelerate the query performance of the blockchain.
3. Privacy information protection: data related to drugs contain much privacy information, which the method proposed in this paper does not consider. Therefore, we expect to implement an attribute-based signature encryption mechanism in the smart contract to further protect privacy information.

## Abbreviations

API: application programming interface
DAPP: decentralized application
DL: distributed ledger
DLT: distributed ledger technology
DoS: denial of service
EHR: electronic health record
EMR: electronic medical record
IDE: integrated development environment
IPFS: InterPlanetary File System
PoW: proof of work
SFDA: State Food and Drug Administration
RFID: radio-frequency identification
RSA: Rivest-Shamir-Adleman
TCM: traditional Chinese medicine
